# Analecta

**Published:** 1826-10-01

**Authors:** 


### ANALECTA,

OR

### MINOR PERISCOPE.

" Ore trahit quodcunque potest, atque addit acervo."

1. *Stethoscopic Indications of the Pregnant State.* Among the various useful purposes to which the Stethoscope has been applied since the first publication of the invention, one of the most important is its employment to ascertain the pregnant state. The merit of discovering its utility in this way is due to M. Lejumeau de Kergaradec, the friend and "compatriot" of Laennec, who published a small *brochure* on the subject in 1822.* A few additional particulars and some new observations, respecting the indications it furnishes, are given in the recent edition of Laennec,† from which we extract a succinct account to lay before our readers.

The stethoscope, applied to the abdomen, affords two distinct indications, which may be regarded as the most unequivocal signs of pregnancy we possess. The *first* of these is the *action of the fœtal heart.* The *second* is a *simple pulsation accompanied with a rushing noise,* designated as the *noise of the placenta* (bruit placentaire) by M. Kergaradec, because its seat is in the placenta, or in that part of the uterus to which the placenta is attached.

(1.) The *Action of the Fœtal Heart* is characterized by *double pulsations,* which are always much more rapid than in the adult subject, being generally twice as frequent as the maternal pulse. These pulsations are distinctly audible from the sixth month of pregnancy, and are sometimes heard a little earlier. Their *situation* is mostly extensive, varying with the position of the child, and being often perceptible in a space a foot long, and three or four inches broad. The precise point whence the pulsations arise, may be always ascertained by the intensity of the noise, which increases or diminishes according as we examine nearer or farther from their seat. It is probable that the space over which the fœtal heart is perceived will be greater, in proportion as the child is placed closer to its membranes, and, consequently, in proportion as the liquor amnios is small in quantity.

The noise sometimes ceases to be audible for hours or even days together; Laennec thinks this may sometimes depend upon the feeble action of the heart, but more frequently is owing to the child having changed its position; so that no part of its trunk touches the membranes, or because a fold of intestine has intervened between the uterus and abdominal parietes.

The action of the fœtal heart is a sign respecting which there can be no uncertainty, nor can it be mistaken for any other; for, although we sometimes hear the heart of the mother, when examining over the epigastrium, sides, or loins, the extreme *difference of frequency,* between the pulsations of the child's heart and those of its parent, prevents the possibility of error in this respect.

* Entitled *Mémoire sur l'Auscultation appliquée a l'Etude de la Grossesse,* par M. Lejumeau de Kergaradec, D. M. P. Paris, 1822. 8vo.

† Tome ii. pp. 457. 466.

The state of the circulation in the fœtus is not affected, or, at least, not constantly affected, by agitation in the maternal circulation, and *vice versâ*. Once, whilst M. Kergaradec was examining the pulsations of the fœtal heart, they were suddenly accelerated so much, that it was impossible to count them: the mother was in a perfectly calm state at the time, and her pulse was not at all quickened. In the course of a few seconds the fœtal pulsations resumed their accustomed frequency, which ranged from 120 to 160. It has also happened to Laennec to witness the fœtal heart suddenly acquire extraordinary energy, its *noise* becoming almost as great as the healthy adult heart; but not accompanied by *impulsion*, or any notable change in the *rhythme* or the *frequency* of its strokes. The phenomenon lasted only a few seconds, and the mother experienced no kind of emotion.

(2.) With regard to the second phenomenon, *the noise of the placenta*, Laennec remarks that it is "evidently an arterial pulsation perfectly isochronous with the pulse of the mother, and accompanied by a rushing noise," resembling the blast of a pair of bellows. There is no sensation of *shock* or impulsion along with this pulsation: it is *heard* only, and it appears to be too deep-seated to be *felt*. The place which it occupies never changes, but it varies in different individuals, and is seldom so large in extent as the space in which the fœtal heart is perceptible. It does not commonly surpass three or four square inches, but sometimes extends over a space exceeding the size of the hand. On one occasion, when visiting the Hôpital de la Maternité, M. M. Laennec, Kergaradec, and Lens, found a subject in whom it was perceptible throughout the whole of the right lateral region of the abdomen (flanc droit) and the right side of the loins.

The *time* when the pulsations of the placenta commonly begin to be heard, is the fourth month. As soon as the fundus of the uterus has risen above the upper brim of the pelvis, so that it can be brought into contact with the abdominal parietes by the pressure of the extremity of the stethoscope, the noise may be heard very distinctly, and sometimes it is even louder than at the full term of pregnancy. At this period, too, its character is peculiar, and different to what it is afterwards. It seems as if a rushing noise, of a somewhat whistling or sibilant nature, *resounded* in an empty bottle; whilst, at a more advanced period of pregnancy, it is almost invariably dull, very diffuse, and gives no sensation of being limited to the calibre of an artery.

This indication, like the action of the fœtal heart, is not continually present; there are some days when it can scarcely be detected; and it often disappears and reappears during the time that we are making our examination.

That the seat of the phenomenon is in that part of the uterus to which the placenta is attached, has been proved by M. Ollivry, a physician residing at Quimper, who, by introducing his hand immediately after the expulsion of the fœtus, in four cases, found the situation of the placenta to correspond with the place where the noise was audible previous to delivery. The same person has also pointed out another strongly corroborating fact, viz. its disappearance immediately, the moment that the umbilical cord is divided. Laennec does not consider it arises from the placenta itself, but from that branch of the artery by which it is principally supplied. We shall have occasion to examine the facts adduced in support of this opinion in our review of the auscultic indications of the heart.

The noise of the placenta and action of the fœtal heart are commonly found on opposite sides of the body: this, however, is not constantly the case; for sometimes both the phenomena are audible on the same side, and in one instance M. M. Laennec and Kergaradec perceived the heart's action

*behind* that of the placenta, the place at which they were examining being the anterior part of the hypogastric region.

In cases of *twins*, Laennec suggests, that there would be *two hearts*, and, perhaps, also, the pulsations of *two distinct placentas*, perceptible in different places. He has known one instance of this kind detected by the stethoscope previous to delivery. He also conceives that it may assist us in determining the *position* of the placenta, and of the fœtus, and, perhaps, throw some light upon extra-uterine pregnancies.

The study of these phenomena requires much greater attention than the indications of thoracic diseases. The noises being very slight and feeble, it is necesssary that the apartment be perfectly still. On account of their intermittent nature, we are sometimes obliged to spend a considerable time in our examinations, and to repeat them several times; and it is particularly requisite to be well acquainted with the character of the indications themselves, so as to distinguish them from any others that may be present; particularly from the action of the mother's heart;—from a dull noise, like the passage of air through thickish fluid, which the peristaltic action of the intestines causes; and, also, from the noise occasioned by the contraction of the observer's own muscles, in consequence of the pressure he is obliged to use in applying the cylinder, in order to keep the uterus and the parietes of the abdomen in apposition.

*Remarks.* The advantages of these indications over the usual methods of ascertaining the pregnant state must be too obvious to every one to require demonstration. As they depend upon the presence of a fœtus and a placenta, when they have once been heard, no doubt can exist upon the subject. The only material disadvantage attending them, that we perceive, is, that the inverse of this proposition does not hold good, on account of the phenomena not being always present or discoverable; so that their *non-existence* is not equally conclusive of there being *no fœtus*, as their presence is of its existence. Judging, however, from our own investigations, we apprehend that an attentive observer will rarely have to repeat his examinations many times, without finding one or other of them present, in subjects really pregnant; and, therefore, we conceive that he would be warranted in regarding the reverse as extremely probable, if he had repeatedly obtained negative results only.

The *objections* likely to be urged against the adoption of this method that occur to us, are, the indecency of exposing the female abdomen, the difficulty of acquiring a competent knowledge of the phenomena, and the liability of mistaking them. With respect to the first of these it is only necessary to observe, that so long as women can be induced, for the sake of alleviating suffering, or by other reasons, to submit to the examination *per vaginam* usually resorted to, no objection can apply against an examination, so infinitely less repugnant to the feelings, as that by the stethoscope. The difficulty of acquiring the use of the stethoscope, were it not frequently urged, would scarcely merit attention. Every thing has its difficulties, and auscultation has its full proportion: but they are not insurmountable to those who wish to learn, and are willing to bestow sufficient time and attention upon the study. To obtain practical skill in applying the stethoscope is not so difficult an acquisition as to play a musical instrument; and its sounds are much more distinguishable from each other than those of music. Attention, patience, and perseverance, are the three most important qualifications in the candidate for stethoscopic celebrity. The assertion which some individuals have put forth, that in their examinations they have not been able to discover any indications, may be answered by informing them, that *others* have detected them, and found them of great practical utility. Whether their want of success arose from defects in the manufacture of the instrument they employed, neglect of the necessary precautions, an imperfect manner of applying it, want of attention, or personal defects in the auscultative organ, is for them to determine. The student will be in little danger of mistaking the auscultic phenomena of pregnancy, if the characteristic distinctions above-mentioned are carefully attended to. We think Laennec has rather *over-rated* the difficulties attending the examination of these indications than otherwise.

We learn from Mr. Jowett, of Nottingham, whose stethoscopic researches we have several times had occasion to refer to in the pages of this Journal, that he has observed the motions of the fœtus to be much more perceptible through the medium of the stethoscope than by the hand alone. The following notice respecting the respiration of the new-born infant has been communicated from the same quarter.

---

2. *On the Establishment of the Respiration in the New-born Child.** In a paper inserted in the Mémoires de l'Académie des Sciences, for 1769, Portal advanced an opinion that the right lung, in the new-born infant, respires before the left. He also adduced some facts to shew that the inflation of the latter does not take place until some time after birth.†

In Paris and Fonblanque's Medical Jurisprudence these opinions of Portal's are regarded as established facts; and further, we find a statement that a physician to a foundling hospital on the continent, who had been daily accustomed to examine the bodies of the infants which died soon after birth, had generally found only one or two square inches of pulmonary structure, crepitous or permeable to air: from which fact it is inferred that the complete inflation of the lungs does not take place immediately after birth; but that the respiration is established in a *gradual* manner.

Soon after I had commenced the use of the stethoscope, it occurred to me that the correctness or incorrectness of these facts might be satisfactorily determined by examining the respiration immediately after birth by means of the cylinder. I accordingly made the experiment, and the result of repeated examinations has uniformly been, that *vesicular respiration* (or the respiratory murmur occasioned by entrance of air into, and its expulsion from, the air-cells) is audible *throughout both the lungs*, as soon as the child has made its first inspiratory efforts.

This experiment proves that the function of respiration is completely established immediately after birth; and therefore controverts Portal's ingenious hypotheses, and the opinions drawn from the appearances the lungs present after death.

I am not disposed to question the correctness of the statement, that there is seldom more than a small part of the lungs found to be crepitous or pervious to air, as it accords with my own observations in most of the opportunities I have had of making such investigations. But it appears to me, that, in the new-born infant, as in the adult, a considerable extent of pulmonary structure that has once been inflated in the act of respiration, may again be rendered impervious to air, by disease or by changes which

---

* Mr. Jowett, in a letter to the Editor.

† The paper in question is reprinted in Portal's Collection, entitled, "Mémoires sur la Nature et le Traitement de plusieurs Maladies, Tome Ier. pp. 29-43. (Paris, 1808, 8vo.)

take place in the dying moments: and, for want of attending to these circumstances, I think an error has been committed, in regarding the non-crepitous portions of lungs (that have evidently respired) as not having been inflated. We frequently observe inflammatory engorgement, hepatization, tuberculous infiltration, œdema, sanguineous engorgement caused by obstruction in the circulation, &c. rendering a great portion of the lungs so dense as to sink in water; and some reasons incline me to regard the last of these states as a common cause of the appearances observed in new born children.

---

3. *Important Toxicological Experiments*.* The word antidote was formerly employed to designate the substances supposed to possess the property of counteracting in the animal economy the deleterious effects of certain poisons; but, of late years, toxicologists not finding any of these precious medicines stand the test of experiment, have restrained the acceptation of the word, and applied it only to such chemical agents as are capable of rapidly decomposing or combining with the poison, and of transforming it thus into a new compound, the action of which is no longer dangerous. By substances, possessing these qualities, it is impossible to act upon the poison that has already been absorbed, or to diminish the effects it may already have produced on the digestive canal, &c. but one may hope to render innocuous that portion of the deleterious substance still remaining in the stomach, and to preserve life by putting a stop to the action of the poison before it has continued long enough to produce fatal effects.

It is in this manner that, according to Messrs. Dumas and Milne Edwards, the new method of treatment communicated to the Philomatic Society of Paris, by those gentlemen, on the 19th of August, may be productive of the most beneficial effects in numerous cases of poison. It is founded on the known innocuity of metallic copper, mercury, lead, &c. many of the compounds of which are most deadly poisons; and, on the property which iron possesses of decomposing the salts of these metals, and of reducing their bases. Thus, when a piece of iron is placed in a solution of acetate of copper, for example, the salt is quickly decomposed, and copper in a metallic state deposited on the surface of the iron.

As yet the efficacy of this substance has only been tried by Messrs. Dumas and Milne Edwards in cases of poison by verdigris, carbonate of copper, acetate of copper, sulfate of copper, and corrosive sublimate, but it is probable that it will be found equally useful in many other cases. All toxicologists agree that 12 or 15 grains of acetate of copper always destroy a dog in the space of a few hours, provided the œsophagus be secured so as to prevent the poison from being rejected. In the experiments of Messrs. Dumas and Milne Edwards, from 15 to 50 grains of that compound were administered in the same manner, and about an ounce of iron filings injected into the stomach before or immediately after the symptoms denoting the deleterious action of the poison had begun. The œsophagus was then tied as in the former cases, but the animals instead of expiring in the space of 3 or 4 hours were, in general, by that time, pretty well recovered from the effects of the poison, and survived 5, 6, 8 days or longer; so that their death was evidently occasioned only by the ligature of the œsophagus. In one experiment the ligature was taken off the day after the poison had been given,

---

* On the use of iron as an antidote for most of the poisons belonging to the class of metallic salts. By Dr. H. Milne Edwards and Mr. J. Dumas Professors of Chemistry.

and the wound closing sufficiently to permit deglutition, the animal recovered perfectly, and 16 days after was made use of for another experiment. After the administration of verdigris, carbonate, and sulfate of copper, the use of iron filings was equally successful; but, with the last of these salts, it was found that the addition of a small quantity of vinegar rendered the action of the antidote much more certain. The same substance administered in the way we have just mentioned, equally hindered corrosive sublimate given at the dose of 12, 15, or 18 grains, from producing death, as is constantly the case when given alone.

The experiments of Messrs. Milne Edwards, and Dumas, not being as yet published, we have not been able to lay before our readers all the details necessary to enable them to judge of the practical use of this new method of treatment in cases which unfortunately so often baffle the skill of the physician; but the importance of the subject induced us to insert the above communication, and as soon as it is in our power, we will return to the subject and examine it with all the attention it deserves.

---

4. *Curious Neuralgic Affection.* In the Journal Général for April, there is a singular case of nervous affection detailed by M. Hellis, of Rouen, which we shall present in an abridged form to our readers.

A young man, æt. 15, who had been for nine years employed in a stable, applied, in 1822, to M. Hellis for advice. His complaint had commenced some years before with a weary pain near the last dorsal vertebra, which soon spread to the epigastrium, attended with hiccup. This continued for about two years, when this nervous sensation did not confine itself to the back and epigastrium, but shot through the chest, belly, legs, and down to the toes. It particularly affected the upper extremities, passing in the course of the nerves to the tips of the fingers. What is important is, that, on closing the hand, the hiccough and this sensation would cease, but on extending a single finger it would reappear, and, quick as lightning, traverse the parts mentioned above. The intervals of exemption from the paroxysm were various, sometimes weeks, sometimes months. Our author invited M. M. Vigné, Blanche, and Godefroy, to examine the case. They found the patient apparently of robust constitution, appetite good, sleep sound. At the bottom of the dorsal region they discovered a small depressed cicatrix, of which the young man could give no account. During the attack, they observed the diaphragm strongly and spasmodically contracted, accompanied by a kind of convulsive sob or hiccough. They, likewise, satisfied themselves of the passage of the neuralgia from the back to the epigastrium, chest, and extremities, and of its instant cessation on flexion of the leg and hand. The affection resisted all the remedies employed, and the patient quitted the country, in order to study for the Church at Rouen. Here, however, the seclusion, restraint, and fear of being incapacitated for his profession, aggravated the complaint, and brought on fits of melancholy. Under these circumstances he was sent back into the country, and then, after a year's suffering, he gradually recovered, his health improved, and the neuralgia disappeared.

---

5. *Experiments on Pulmonary Exhalation.** The great rapidity with which aeriform and certain volatile substances, when introduced into the veins of a living animal, are expelled by pulmonary exhalation, is a fact fully

---

* By Messrs. Breschet and H. Milne Edwards, M.D's.—*(Private Correspondence.)*

established by daily observations and by the researches of some French physiologists. In the paper read by Dr. Milne Edwards at the Academy of Medicine of Paris, the 26th of July, the authors relate the experiments they performed, in order to ascertain whether the suction which accompanies each dilatation of the thorax is not the principal cause of this exhalation being so much more rapid in the lungs, than in the other parts of the body; on the same principle as we find, by the interesting researches of Dr. Barry, that absorption is most materially influenced by pressure.

In one experiment, Messrs. Breschet and Milne Edwards injected some camphor, dissolved in alcohol, into the abdomen of a dog, and, four minutes afterwards, found the breath of the animal strongly impregnated with both these substances. They next repeated the same experiment, but with this difference, they introduced a tube into the trachea, laid open the thoracic cavity, and kept up respiration by means of a bellows. The mechanism which makes the thoracic cavity resemble a suction-pump, was then destroyed; and, although the artificial respiration was kept up during an hour, there was no smell of camphor or of alcohol in the air expired from the lungs; at the same time, however, a cupping-glass being applied to the exterior surface of the muscles of the abdomen, the smell of camphor soon became perceptible in that part. In a third experiment, essential oil of turpentine was thrown into the arteries of a dead animal, and, on opening the thorax, the abdomen, &c. the exudation of that substance was found to have taken place in all these parts. By injecting a small quantity of essence of turpentine into the femoral vein of a dog, the thorax of which had been previously laid open as in experiment No. 2, and the respiration carried on in the same manner, the exhalation of turpentine took place equally in the abdominal and pulmonary cavities. But when the same operation was performed on a dog of which the thoracic pump was left entire, the essence escaped with much greater rapidity by the pulmonary exhalation, and no trace of it could be detected on the surface of the peritoneum.

From these experiments, and from some others of the same tendency the authors draw the following conclusions.

1. Those substances which do not pass easily through animal tissues by imbibition, after having been introduced into the circulating medium are no longer exhaled by the lungs as soon as the action of the thoracic pump is destroyed; whereas, those same substances would be soon completely expelled by that means if the suction produced by the dilatation of the thoracic cavity was allowed to continue as usual.

2. That when substances which pass through animal tissues with great facility, (as is the case with essence of turpentine) are mixed with the blood they are equally exhaled in all the parts of the body abundantly supplied with blood-vessels, provided the thoracic pump be destroyed; but that when, as in the usual state, the suction produced by the action of that organ is uninterrupted, the exhalation takes place only in the parts subject to the influence of that power.

3. That it is, consequently, the mechanical action above alluded to, which occasions the rapid exhalation by the pulmonary surface of water, alcohol, camphor, essence of turpentine, and Leven's gases introduced into the circulating medium.

In a subsequent paper, Messrs. Breschet and Milne Edwards intend examining the influence of that action on the other phenomena of respiration.

---

6. *Transfusion*. Mr. Brigham, of Manchester, has added another case to those already published, of transfusion of blood in uterine hæmorrhage.

The patient was a woman 40 years of age, who was easily delivered of her eighth child, the placenta being expelled in a quarter of an hour afterwards. In the course of three hours, the patient complained of a violent pain in her back, and appeared slightly convulsed. This was followed by a sudden gush of blood from the uterus, which must have continued more or less for some hours. Four hours after the accident Mr. B. visited the patient, and found her senseless, pale and exhausted, with cold extremities and surface, the pulse scarcely perceptible. The flooding had now ceased. Light nourishment, and stimulants were administered; but without producing the least sign of benefit, or of returning animation. In another hour she appeared to be sinking. Her breathing was short and hurried—face deadly pale—pulse quite imperceptible at the wrist—and, in short, "life appeared to be ebbing fast away." Transfusion was now the only resource, and it was performed in the presence of several medical gentlemen of Manchester. Two ounces were first injected by means of a common syringe, without any sensible change in the pulse or otherwise. But after the third charge had been injected a few minutes, the pulse was bettered (or we should say became perceptible) and the countenance appeared more animated. The transfusion was continued, with intervals of ten or twenty minutes, until ten or twelve ounces of blood were injected. A decided improvement was now evident, and the patient was able to speak, which she had not done for six hours before.

A curious phenomenon is here recorded by Mr. Brigham, which is worthy of notice. Immediately after each injection of blood, the pulse, feeble as it was, appeared to faulter still more, and to become more oppressed; but after a lapse of five or ten minutes, it recovered its beat, became more firm, and evidently demonstrated the effect of the transfusion. On visiting the patient next morning, she was found in a composed sleep, which had lasted four hours. She gradually recovered completely.

In this, as in most of the other cases, there is an opportunity for the sceptics to aver that life would, in all probability, have been preserved had the transfusion never been used. It is impossible to argue against such objections—objections which might be urged against every remedial means, where recovery is the result.—*Ed. Journal of Med. Science.*

Still more recently the operation of transfusion was performed on a female, a patient of Mr. Jewel's at the Middlesex Infirmary, but without success. The woman was of delicate constitution, and exhausted by a severe uterine hæmorrhage. After various means had been employed to recruit this patient, transfusion was had recourse to. Mr. Boyle opened the right external jugular vein, no vein being discoverable in the arm, and about four ounces of blood were thus introduced. No good effect was apparent, and the patient soon expired. As it was suspected that, by possibility, some air might have got into the jugular vein, notwithstanding the care that was taken to prevent it, an examination was made after death, and a very small quantity of air was found in the right cavities of the heart. It is not certain whether this small quantity was introduced during the operation, or generated by putrefaction after death. The accident, however, which happened to M. Dupuytren, on opening the external jugular vein of a patient, and the recent experiments of Dr. Barry, render it probable that transfusion through this channel must be a very hazardous operation, and ought to be avoided, if any other vein can be found for the purpose.

---

7. *Fatal Placental Presentation.* Mr. James, of West-Bromwich, Birmingham, has stated a melancholy case of this kind, in the Repository for

September, 1826. The woman was seven months gone in pregnancy, when a uterine hæmorrhage occurred, after carrying a heavy weight of coals on her head. Mr. James was summoned on the 29th July, 1826, on account of this accident. She was said to have lost three pints of blood. She was not faint—pulse 90, and firm—os uteri admitted the point of the finger, but nothing unusual could be detected. Quietude in the horizontal posture—cold to the labia pudendi—sulphuric acid and opium every four hours. After the third dose, she was seized with a most profuse hæmorrhage. A midwife was brought, who sent for Mr. James, but he did not arrive till many hours after the loss. The woman was apparently then in a dying state. Æther and brandy were exhibited, and a temporary revival followed. Mr. J. found a large knuckle of placenta in the vagina, the os uteri almost fully dilated. He discovered the dense expansion of the placenta implanted directly upon the os uteri in its thickest part. He passed his hand up on one side, and attempted to thrust his finger through the weaker edge, but failed. He then perforated the centre of the mass with his fingers, seized the feet of the child and brought them down into the vagina, giving exit to a pint or more of liq. amnii. A pain occurred then, but did not return. Hæmorrhage supervened. The child's body was extracted, but the head was obliged to be opened, and extracted with a hook. Contraction of the uterus was solicited by the introduction of the hand, keeping pressure on the abdominal aorta, to lessen the distance of the heart's circulation—but all to no purpose—life was gone.

Mr. James suggests the expedient, in profuse uterine hæmorrhages, of placing tourniquets on the limbs, and keeping pressure on the abdominal aorta, until contraction of the uterus takes place. "In this way the heart, brain, and lungs would receive an adequate supply of blood for the sustentation of life." We were not aware that "it is a common practice among midwives to tie ligatures on the limbs, to stay the loss of blood." We did not give them credit for so much philosophy. Many of our readers must recollect that tourniquets used to be applied to the limbs in the cold fit of agues, for the purpose of shortening that disagreeable stage, which it certainly did. We have tried the experiment many times, some twenty years ago. The rationale appears to be, that it shortens the round of the circulation, and keeps the great arteries fuller than they otherwise would be. This expedient is worthy of trial, in such deplorable circumstances.

---

8. *Imperforate Rectum.* A child, nine days old, was lately brought to Bartholomew's Hospital, the abdomen so distended that the convolutions of the intestines could be distinctly seen. On examination, Mr. Earle found that the anus was perfectly formed, but that, at an inch and a half from the external opening, a septum went across, and thus prevented all egress of fæces. A trochar, of large size, was pushed through, and gave vent to a large discharge of flatus and fæculent matters, after which the cannula of the trocar was left in for two days, and then replaced by an elastic gum tube. The passage was subsequently enlarged by a bougie, and the child perfectly recovered.

---

9. *Fatal Dissection Wound.* Another medical man has lately fallen a victim to this terrible accident. M. Gerard, Jun. Professor at the Veterinary School of Alfort, pricked the little finger of his left hand, while dissecting the body of a man who died the day before of putrid fever. A small vesicle, encircled by an inflammatory areola, was first perceived. This was opened with the point of a lancet, and some sero-sanguineous fluid discharged. Presently the whole arm swelled. M. Breschet was called in, and found the patient in a state of great inquietude and suffering. He had had leeches applied, and was bled from the arm, which produced protracted deliquium. The finger was swelled, œdematous, and emphysematous. The arm presented a tumefied and marbled appearance. Caustic was applied, and afterwards emollient fomentations and poultices. This was followed by considerable mitigation of the symptoms, but the patient was haunted with fears as to the issue of the case, and the inflammatory symptoms rose again. Leeches, fomentations, and anodynes were reiterated. The morbid sensibility of the arm was such that the slightest pressure occasioned the most poignant distress. Pain was also complained of in the epigastrium and abdomen. The tongue was covered with a white crust, the edges being red—skin hot and moist—pulse frequent—cerebral excitement great, but without delirium—strong conviction in the patient's mind that the disease would prove fatal. Recamier, Dupuytren, and several eminent men now joined in consultation. Leeches were clustered on the abdomen—and musk with other sedatives were administered internally. At this time a most exquisite pain was felt in the left knee and shin bone, though no redness or swelling could be perceived. Soon, however, some obscure fluctuation was felt, and an incision being made through the fascia of the leg, a small quantity of puriform fluid escaped. Between the twelfth and fourteenth days a still more alarming train of phenomena was developed. Severe pain in the head—intolerance of light—great irritability—and sub-delirium—frequency and irregularity of the pulse—sleeplessness—subsultus tendinum, &c. set in, and these symptoms progressively increased, with a dysenteric state of the bowels. Warm baths calmed a little the violence of these phenomena; but they were always exasperated by a subsequent re-action. The respiration now became embarrassed, and the patient sunk, after a violent and agonizing struggle of 48 hours.

*Dissection.* Fluid between the arachnoid and pia mater—brain and cerebellum firmer than natural—an ounce of yellow serum in the ventricles—but no extraordinary injection of the cerebral vessels. In the chest, about eight ounces of dark-coloured fluid were found on each side, which appeared to be pure blood, or blood mixed with a small quantity of serum. The lungs were remarkably pale, but otherwise not diseased. No other lesion in the thorax. In the abdomen, the peritoneum was pale and healthy. The mucous membrane of the stomach was rather injected in some places, and softened. The duodenum presented throughout its whole extent a sub-mucous emphysema, its lining membrane being raised in small phlyctenæ. The mucous tissue of the jejunum was also elevated by a gazeous fluid—that of the ileum was red with capillary injection, especially about the ileo-cæcal valve. There was no other particular appearance, except in the blood, which was in a completely liquid state throughout the body.

We think it will hardly be doubted that a virus was introduced into the system in this case, though certainly the state of mental despondency which M. Gerard evinced, and which is but too often evinced by medical men labouring under severe disease, gave a fearful activity to the poison received during dissection. It is very doubtful if the caustic and the innumerable leechings did much good in the case just detailed. But on this point our readers will judge for themselves.

The same author, M. Gendrin, has made some experiments on animals, with blood and other fluids taken from diseased subjects, of which it may not be uninteresting to take some notice in this place.

---

*Exp.* 1. An ounce of blood, taken from the vein of a man who was ill and died of a putrid fever; was injected into the cellular membrane of the thigh of a cat. For an hour and a half there appeared to be no inconvenience produced. In 20 minutes more, trembling of the under lip and nausea took place—at the end of two hours vomiting, first of yellow bile and afterwards of green. The animal lay down and uttered some plaintive cries—the breathing was embarrassed. At the end of six hours there were unequivocal symptoms of fever—and in fifty minutes more the animal expired, with slight convulsions, and in a state of extreme prostration. The gastro-intestinal mucous membrane was healthy, but that of the trachea and bronchia was red—lungs contained black blood—and the same was found in the arteries. Two ounces of black blood were effused in the cavities of the pleura. No morbid appearance in the brain or spinal marrow.

*Exp.* 2. Some blood which had issued from the nose of the same fever patient was injected into the crural vein of a small dog, and, four hours afterwards, the animal vomited green bile—was depressed, with quick pulse, &c. In seven hours he died. On dissection, the blood was found to be black and fluid in all the vessels, with a red state of the bronchial mucous membrane.

These experiments are confirmatory of those made some years ago by M. Gaspard, where the injection of pus and other secretions into the cellular membrane and vessels of animals caused their death. They also support the idea that, in certain fevers, some poisonous miasma is received into the circulation, and gives rise to the terrible phenomena that result. What but an aerial poison could produce the destructive fevers of Batavia and other deadly situations that have been the graves of so many Europeans?—*Rev. Med. Avril*, 1826.

---

10. *Infanticide.** Information having been given to the county procurator-fiscal that one Margaret Patterson had been delivered of a natural child, which, from certain marks and other circumstances, was supposed to have suffered violence causing its death, Dr. Grace and Dr. Scott were ordered to examine and report upon the case. They were informed that the female had been delivered, without assistance, the preceding day—and that the child was found dead by the neighbours, under suspicious circumstances. The child appeared full grown, and free from putrescency, weighing six pounds, two ounces, and measuring 19 inches in length. The umbilical cord appeared to have been torn, and was untied, except by a few threads loosely thrown round it. The face was slightly livid, eyes turgid, slight puffiness above the ears, but no injury of the integuments, permanent deep indentation encircling the neck, bordered by a stripe of rose-red discoloration. There were similar marks on each side of the thyroid cartilage, with abrasion of the cuticle. Under these discoloured marks, there was injection of the capillaries, especially over the cartilages of the trachea, which were compressed and injured. The chest was round and prominent—lungs fully dilated—and swam in water—large blood-vessels at their roots, filled with dark-coloured blood—ductus arteriosus empty—venæ cavæ and right auricle contained blood—viscera of the abdomen healthy—urinary and gall-bladders empty—large coagula of blood under the integuments of the head covering the parietal bones—pericranium vascular, and separated from the bones—parietal bones fractured, one into five, the other into three pieces—surface of the brain vascular, and covered with coagulated blood—coagulated blood

---

* Dr. Scott and Dr. Christison. Ed. Journal, July, 1826.

in the ventricles. There was meconium on the shawl in which the child had been wrapped.

Such were the anatomical and physiological proofs of infanticide—and we think they could not well have been stronger. The moral or circumstantial evidence was nearly as conclusive as the physical. The mother had concealed her pregnancy—made no preparation for the birth of her infant—and secreted it under the chaff-bed on which she lay. When detected, she made a full confession of the whole affair, but still denied having committed the crime of infanticide. She averred that she did not think herself in the family-way, though she had borne a child previously. She said the infant was born with the umbilical cord twisted round the neck, and that it never cried or was heard to breathe. It is needless to waste time in refuting these assertions; yet, so much afraid was the procurator-fiscal of the failure of all this medical and moral evidence before a legal tribunal, that he abandoned the greater charge of infanticide, on the mother's pleading to the minor offence—the concealment of pregnancy. She, accordingly, escaped punishment. If the graver charge had been insisted on, the plea of insanity was intended to be set up by her counsel. Convinced as we are that, in nine cases out of ten, the act of suicide is owing to temporary insanity, we are strongly disposed to extend the same veil of mercy to infanticide. The female wild animal—the female savage—and even the civilized and selfish mother will sacrifice their own lives in defence of their offspring. When, therefore, we see so deep-rooted an affection torn up and reversed; we have strong cause for believing that REASON has been previously shaken from her seat—and that the wretched mother has been hurried along by the impetuous tide of her feelings and her fears, to commit that, from which she would have recoiled with horror, under a medium equanimity of her moral nature. This doctrine may be stigmatized by the rigid moralist, as dangerous or even false—but we hope it will always be that to which a feeling judge and intelligent jury will lean.—*Ed. Journ. July* 1826.

---

11. *Extra-uterine Conception.* A case of this kind is related in Majendie's Journal for January, by Dr. Doudement, of Rouen. The particulars are as follow.

Mad. B. æt. 34, of a good constitution and active disposition, complained in 1823, of dysury, pain in the kidneys and epigastrium, and discharge from the vagina. Under the use of warm-baths, emollient injections, &c. these symptoms disappeared. Towards the middle of May, 1824, the lady suffered from fits of nausea, which disappeared in a few days; the menses ceased, and the breasts swelled, giving rise to the supposition that she was pregnant. The menstrual discharge, however, soon reappeared, but ceased in the course of a few hours. From this time there used to be every day a discharge of blood from the vagina; this discharge was neither abundant nor of long duration. The patient preserved her embonpoint, and was in the habit of walking a good deal. No examination being permitted, Dr. D. attributed the symptoms to irritation of the mucous membrane of the uterus or perhaps to a commencing polypus; he, therefore, enjoined strict repose, cooling diet, and the application of leeches to the groin. The bleeding from the leeches, though inconsiderable, caused some degree of weakness, and loss of flesh was perceptible. The husband now thinking that pure air would do more good than the doctor, took his lady, in the beginning of July into the country, but though her health seemed improved, still the discharge of blood continued, with general malaise, and they were soon obliged to return. Dr. Gosseaume being consulted, thought there was nothing serious in the complaint, and prescribed only absolute rest. The symptoms began to disappear, but Mad. B. taking active exercise again, they returned accompanied with dry cough, and heat of skin, particularly at nights. On the 5th August, after using some exertion, an abundant discharge of blood from the vagina, took place, with violent vomitings; face pallid, limbs stiff; pulse small and frequent; and severe darting pains in the right hypochondrium, relieved somewhat by the sitting posture. Opiate draughts, diluents, fomentations to the abdomen and legs, and frictions were employed during the night, and by the next morning all the symptoms were much mitigated. In the course of a few days the lady recovered her health, soon again to lose it. An examination was now allowed, and Dr. D. found the neck of the uterus elongated and descending to within an inch of the orifice of the vagina, the uterine orifice round, its edges granulated and hard; the uterus developed as in the second month of pregnancy; the belly was exquisitely sensible. A physician, who was consulted, gave his opinion that it was disease of the uterus and ovaries; M. Dupuytren was applied to, but gave no decided diagnosis. The poor lady's health now visibly declined. Another attack of her former symptoms came on; on examination there was found retroversio uteri; some feeble contractions of the organ took place; the pain, weakness, smallness of pulse, coldness of the extremities increased, and she died in agony on the 12th September.

*Dissection. Abdomen.* A semicircular incision being made from one hypochondrium to the other, about three pints of mingled blood and serum issued. From the midst of the convolutions of the intestines, a male fœtus escaped, about the size of one three months and a half old; it was discoloured, and, in many places, the epidermis was detached. In the pelvis, between the cæcum and rectum, to which it adhered, appeared the fœtal cyst, dark and of the size of the fist (poing.) In the cyst, to the left, was an opening two inches in length, and it adhered to the omentum, which passed over it and formed a kind of band, keeping it in its place. This cyst was found to arise from the right ovary; its interior was filled with clots of blood, and its lining membrane like that of the amnion. The mucous coat of the uterus was injected, and its internal surface covered by a gelatinous membrane. The peritoneum was reddened. Such are the principal facts of this case.

---

12. *Rheumatism of the Heart cured by Acupuncture.** Mademoiselle H. æt. 18, of a good constitution and nervous temperament, after having lived for several years in a damp house, was attacked with rheumatism of the legs and arms. On her leaving the house this was relieved, but she now began to be seized frequently with acute pain in the heart, aggravated by any sudden emotion, by cold and damp weather, and by changes of temperature; this pain was accompanied by palpitations, and often followed by a kind of delirious loquacity; its duration was from a quarter of an hour to three hours and more. To these attacks, with the exception of the pain and palpitations, the young lady had been subject some years previously, after the loss of her mother. Baths, bleedings, leeches were employed in vain.

On applying to M. Peyron, the narrator of the case, it was found by auscultation that the pulsations of the heart were unusually violent, and distinctly audible at the upper and back part of the left side of the chest, and even of the right; the impulsion of the ventricles was in duration to that of the auricles, as one to five eighths; pulse quick and intermitting.

---

* M. Peyron. Revue Medicale, May, 1826.

It was determined to try acupuncture. A needle, 13 lines in length, was introduced, without, however, touching the heart. This was followed by one of those semi-delirious paroxysms to which the patient was subject. A second needle, 15 lines in length, was used with a similar effect. A needle 18 lines long, was now directed from the upper border of the cartilage of the sixth rib, through the pericardium, until its point entered the heart. This was followed by alleviation of the pain in that organ, nor has it since returned.

13. *Extirpation of an Ovarium.** A back settlement of America—KENTUCKY, has beaten the mother country, nay, Europe itself, with all the boasted surgeons thereof, in the fearful and formidable operation of gastrotomy with extraction of diseased ovaria. In the second volume of this series, page 216, we adverted to the cases of Dr. Macdowel, of Kentucky, published by Mr. Lizars of Edinburgh, and expressed ourselves as sceptical respecting their authenticity. Dr. Coates, however, has now given us much more cause for wonder at the success of Dr. Macdowel; for it appears that, out of five cases operated on in Kentucky by Dr. M. four recovered after the extraction, and only one died. There were circumstances in the narratives of some of the first three cases, that raised misgivings in our minds, for which uncharitableness we ask pardon of God, and of Dr. Macdowel of Danville. The two additional cases now republished (for it appears that the cases were published, though in a very unsatisfactory form, in the American Eclectic Repertory) are equally wonderful as those with which our readers are already acquainted. We shall only glance at one of these.

In April, 1817, Dr. Macdowell removed from a negro woman a tumour of the ovary, weighing five pounds. The ligature, which was passed round the fallopian tube, slipped, and the patient lost a large quantity of blood. Ligatures were then applied to several of the arteries individually; but this also failed, as some of them cut through. "*With much difficulty a large ligature was now put round the whole stump of the tube, and secured by stitching it in and out of the tube at several places.*" This effected the purpose, and the patient recovered of the operation. This was the fourth case. The fifth was unsuccessful.

When we come to reflect that all of the women operated on in Kentucky, except one, were *negresses*, and that these people will bear cutting with nearly, if not quite as much impunity as dogs or rabbits, our wonder is rather lessened, and so is our hope of rivalling Dr. Macdowell on this side of the Atlantic.

In July, 1821, we are informed that Dr. Nathan Smith, of Yale College, extirpated an ovarium from a lady of Vermont. Through a small incision of three inches in extent, the tumour was first tapped, and eight pints of a dark ropy fluid were removed. The sac was then drawn out, and separated with the knife from the omentum to which it adhered. Two arteries in the omentum were tied with *leather ligatures*. The ovarian ligament was then divided, two arteries secured as before, and the ligament returned. "The sac was supposed to weigh between two and four ounces." No unfavourable symptom occurred, and in three weeks the lady was able to sit up and walk. This case is related in the 18th volume of the Edinburgh Medical and Surgical Journal.

* Dr. A. G. Smith. North American Medical and Surgical Journal, No. 1, January 1826.

The still more recent case is by Dr. A. G. Smith, and Danville, Kentucky, is still the scene of operations.

The patient was a negress, who had a tumour in her abdomen of two years standing. Her general health was good, and on the 24th May, 1823, Dr. Smith made an incision from the umbilicus to within an inch of the pubes. The peritoneum was then carefully incised, when the tumour presented itself. A large opening was made into it with the scalpel; and several pints of fluid evacuated, when it so far collapsed that the operator was able, though not without difficulty, to get it out of the abdomen. The attachment to the uterus was found to be not much larger than the broad ligament usually is. The tumour appeared to be an enlargement of the whole ovary of that side. The attachment was surrounded with a strong ligature of white silk—the tumour separated—and the wound was secured by the interrupted suture:—twenty-five drops of laudanum were then administered, and the patient put to bed. As sickness came on, 50 drops more were administered, and shortly afterwards 200 drops were given per anum. All this did not allay the gastric irritability, and, therefore, five grains of opium were introduced as a suppository. We need not pursue the diurnal details. Suffice it, that the negress recovered. The tumour was found to be of a scirrhous nature, and to contain a considerable quantity of bony matter.

Dr. Coates, one of the editors of the Journal from which we have taken this account, characterises "this operation as peculiarly American, seldom, if ever, having been performed with success, in Europe." Dr. Coates has quoted in italics our scepticism respecting the success of this operation, as stated in the second volume of this series, page 408. If the operations in Kentucky be authentic records, we have been wrong in our prognostication most assuredly, and happy we are to find ourselves in error on such a point. We did not know that the cases had been published in the Eclectic Repertory, and, from an observation of Dr. Coates himself, it appears that the *mode* in which they are narrated had "*drawn forth comment*," in the Repertory. It was this *mode* of narration which excited our scepticism, and we must confess it is not yet removed. If we were wrong in our prognostication, we were so in good company—that of Diemberbroeck and Sabatier, who believed the operation to be altogether impracticable. Dr. Coates does not consider the case of Mr. Lizars as one of unequivocal success. We think it is. For although one ovary was left behind in a state of disease, the other was successfully removed, having carefully examined the woman ourselves in London afterwards.

---

14. *Acupuncture.* In the last number of the Medical Repository, Dr Ewing has related a case which exemplifies, in a very striking manner, the surprising and unaccountable effects of this curious operation—or, of the influence of mind over matter, for we scarcely know to which of these causes to attribute the almost miraculous result.

A young lady had been afflicted, at intervals, for eighteen months, with a severely painful affection of the nerves of the right cheek, immediately below the orbit, and extending to the angle of the lower jaw. On the 14th January, 1826, she was attacked more violently than usual, and all remedies failed. Next day, acupuncture was proposed, and eagerly submitted to by the young lady. Three needles were inserted, about an inch from each other—two of them parallel with the inferior edge of the orbit and half an inch below it—the third below, and equi-distant from the others. The first two were introduced to the depth of three quarters of an inch, slowly and with a rotatory motion—the third, to full an inch in depth. The second needle was scarcely introduced, when the patient exclaimed that the pain had entirely left her. When the third was introduced, such was the relief, after previous exhaustion, that she reclined her head on a pillow, and fell fast asleep. There was no return of the pain. Several weeks had passed away, when the report was made, and no attack had been experienced.

---

15. *Congenital Blindness cured at an advanced Age.** When this lady came under Mr. Wardrop's care, she was in her 46th year. The right eye-ball was collapsed, the left retaining its natural globular form. The cornea of this eye was transparent, except at one point where there was a linear opacity, probably the cicatrix of a wound made by a Parisian oculist, in an unsuccessful attempt to restore vision when she was six months old. The anterior chamber of the eye was of its natural capacity, but no vestige of a pupil was perceptible, some streaks of yellow lymph being deposited in an irregular manner over the central part of the iris. There was reason to believe that the retina was sound; for though she could not perceive objects, or form any idea of colours, yet she was able to distinguish between a very light and a very dark room, and between a gloomy day and sunshine. Under this impression, Mr. W. thought the attempt to restore sight by an artificial pupil was worthy of a trial. On the 29th January, he introduced a small needle through the cornea, and passed it through the centre of the iris, but could not destroy the adhesions which had shut up the pupillar opening. After this, she could perceive a little more light, but not forms or colours. On the 8th February, a second operation was performed, by passing a sharp-edged needle through the sclerotica, bringing its point through the iris into the anterior chamber, repassing it into the posterior chamber at a small distance, and then dividing the portion of iris thus included between the two perforations of the needle. A slight inflammation followed—the light became offensive—but it was evident the sight was still very imperfect. On the 17th February, a third operation was performed, which consisted in still further enlarging the opening in the iris, and in removing some opake matter by a needle introduced through the sclerotica. In returning home from Mr. Wardrop's house, with the eye covered with a loose piece of silk, the first thing she noticed was a hackney-coach passing, when she exclaimed—"What is that large thing that has passed by us?" In the course of the evening she requested her brother to shew her his watch, which she narrowly surveyed close to her eye. She remarked, there was a dark and a bright side. She pointed to the hour of 12 and smiled. When asked if she saw any thing more on the dial-plate, she replied "yes," and pointed to the hour of 6, and to the hands of the watch. She then looked at the chain and seals, remarking that one of the seals was bright, which was the case, being of crystal. Next day she refused to look at the watch, observing that she was confused by the visible world thus for the first time opened to her. On the third day, she observed the doors on the opposite side of the street, but mistook their colour. This day she saw the nose on her brother's face, (dimensions and colour not stated,) and on the 6th day saw still better, but was bewildered, from not being able to combine the sense of sight and touch, feeling dis-

* Case of a lady born blind, who received sight at an advanced age by the formation of an artificial pupil. By James Wardrop, Esq. F. R. S. Ed. &c. From the Philosophical Transactions.

appointed in not having the power of distinguishing at once by the eye those objects which she could so easily distinguish from one another by feeling them. In short, she had almost every thing to learn—for although she could distinguish one object from another, she knew not, by the newly-acquired sense, what any object was—nor could she form any idea of its distance from her. She was, however, highly delighted with restored vision, and we almost envy her the pleasure which she must feel on the sight of so many *novelties*, at an age when novelties begin to be very scarce articles with most human beings—even of her sex!

There is no other case on record where the sense of vision was restored at so late a period of life—and it is not a little remarkable, that the optic nerve should remain fit to receive the impressions of external objects for the long space of 46 years, during which, it was totally excluded from the performance of its function. The operation is very creditable to Mr. Wardrop, and we wish him long life and many opportunities of *enlightening*, not only the visual orbs of his fellow creatures, but the dark paths of medical science. We also wish him plenty of "*pupils*," natural and artificial, to operate on in Bartholomew Close—which some folks think is rather *too close* to Bartholomew.

---

16. *Remarkable Disease of the Heart.** 1. The first case of the curious disease in question, was observed by Dr. Young, at St. George's Hospital, in the person of a young woman who had been subject, for ten years, to occasional palpitation of the heart, and sensation of suffocation in the throat, with pain in the left side of the thorax, extending to the back and left shoulder, accompanied by vomiting. Each attack commenced with sense of obstruction at the scrobiculus cordis, rising to the left shoulder, and descending the left arm to the little finger. These paroxysms were at first at long intervals, once in several months, and only of an hour or two hours' duration. Within the last two years, however, they had become more frequent—yet still she was perfectly free from ailment in the intervals.

"Since Christmas last, these attacks have increased in frequency and violence, and she has suffered four weeks, with only one intermission, from the present attack. *Pulse above two hundred,* occasionally too rapid to be counted;† with throbbing of the carotid arteries communicated to the veins,

---

* Drs. Young—Macleod—Otto.

† We never could count the pulse with any degree of certainty beyond 150 or 160 in the minute: we were, therefore, a little surprised at the foregoing statement of Dr. Young. It is not impossible, however, that a profound knowledge of the mathematical and other exact sciences may confer a power of enumerating the pulses superior to that possessed by the generality of the profession. The following is the opinion of one of the most talented physicians this country has produced—the late Dr. Parr. "The pulse may be counted with some degree of accuracy till it reaches *one hundred and forty* strokes in the minute—perhaps, by strict attention, to one hundred and seventy. Beyond, all is rude conjecture."—*Art. Pulsus, in Med. Dictionary.* But, we may further observe that, had we the power of *distinctly* counting the pulse to, or beyond 200 in the *minute,* we do not believe that the heart has the power of *distinctly* throwing out blood to that amount of pulsations. At least we have never felt a distinct pulse beyond the number we have stated above. It is but fair, however, to say, that Dr. Young may quote Wendtt, *(de pulsus mutatione,)* who maintains that he could distinguish 243 pulses in the minute!!

through which the rapidity of the circulation is remarkably visible." She could sleep only on the right side, with her head raised—burning heat was felt over the left side, with a sense of great anxiety at the præcordia—skin cool—no thirst—tongue clean—bowels costive. She had one lucid interval of 24 hours in the hospital, during which the pulse was 90, and the symptoms greatly abated. With this exception, the symptoms continued to increase in violence until within a few days of her death, when she became insensible, with occasional screaming convulsions, œdema of the lower extremities, and incipient gangrene of the feet.

*Dissection.* The lungs were more solid than usual, and contained much watery fluid. The heart was rather large, and flatter than is usual for so early an age. On opening the right ventricle, a solid substance presented itself, resembling coagulated blood; but, on more accurate examination, it was found to consist principally of effused lymph, in masses of various shapes and sizes, and in different states of formation, the colour being mostly yellow. Many of these portions were detached, but others adhered to the texture of the heart. Some of them formed distinct globular cysts, varying from the size of a pea to that of a small walnut. When these cysts were cut open, the inner surface was reticulated, and some of them contained a few drops of pus, while others again were entirely empty. The greater part of the lymph, however, was in solid masses, easily broken or torn by pressure. The lymph was most abundant at the lower insertions of the fleshy columns of the tricuspid valve, but extended also into every hollow and sinus formed by the reticulated texture of the sides of the ventricles. In one place the adhesion was so firm as to make it somewhat doubtful whether or not there was abrasion of the ventricular lining. In the left ventricle there were exactly the same appearances, but on a smaller scale. The brain was natural in appearance, except a softened portion in the left corpus striatum. The circulation in the internal carotid artery of the left side was obstructed by a firmly adherent coagulum which was found within it.

2. Dr. Macleod informs us that he was present at a dissection by the late Dr. Gordon, of Edinburgh, when two cysts were found in the right auricle of the heart—one being flat, and applied over the auriculo-ventricular orifice—the other globular, and the size of a boy's marble. Both appeared to consist of concentric layers of organized lymph, about the thickness of a half-crown piece. The globular one was filled with a dark-reddish fluid—the other empty.

3. To the above we shall add a case related lately by Dr. Otto. The patient was a soldier, who died of disease of the testicle, and who does not appear to have exhibited any symptoms of cardiac affection during life: on dissection, the size and structure of the heart were natural, but the right ventricle was found to contain an immense number of hydatids, which were attached to the eustachian valve by seven slender stalks or filaments, one of which seemed ligamentous. The valve itself was thickened and enlarged, and could not have completely performed its function in consequence of the position of the hydatids. The number of these bodies amounted to between sixty and eighty.—*Journ. Univ. des Sciences Med. Juillet*, 1826.

We have registered these cases as presenting curious and rare specimens of morbid anatomy. The symptoms in Dr. Young's patient, would, of course, have led to the conclusion that there was organic disease of the heart, though the nature of it could hardly have been guessed at.

17. *Injuries of the Thorax.**—*Case* 1. A man was brought to the Middlesex Hospital in the middle of the night, said to have been run over by a carriage. He complained much of his chest, and his breathing was laborious. On pressure, several of the ribs on the left side seemed to yield—and much crepitus was felt on moving the body. The exact number of fractured ribs could not be ascertained, in consequence of the cellular emphysema, rapidly extending over the body. The breathing becoming more difficult, and the pulse rising, an attempt was made to bleed, but little blood could be extracted, and little relief was obtained. The emphysema rising to a great height, the temporal artery was opened, and a large quantity of blood was abstracted, but without any relief. Mr. Bell was then summoned, and immediately made an incision on the upper edge of one of the fractured ribs, punctured the pleura, and enlarged the opening with a bistoury. A forcible rush of air from the chest was the consequence, the breathing was relieved, and the tumefaction of the face lessened. In a few hours the breathing again became laborious, and relief was obtained by clearing the wound of coagulated blood. This operation was repeated at midnight. *Second day.* Breathing easier—cough troublesome—expectoration glairy, with streaks of blood, but no froth. A bandage was applied lightly round the thorax in the morning, with the view of steadying the ribs. This was, however, soon left off in consequence of the increased difficulty of breathing. Purges. Salines with antimony, with tinct. camph. comp. every six hours. Venesection as often as the pulse rose. Next morning he was better, but he changed for the worse and died in the evening.

*Dissection.* Ten ribs on the left side were fractured; three of them projected into the cavity of the chest, in which there was much blood and serum. A laceration was discovered in the lung. Bronchiæ inflamed—lungs gorged with blood—old pleuritic adhesions.

*Remarks.* Mr. B. observes that emphysema is caused either by the rib being pressed in upon the lung, or from the lung itself being abraded by the motions of the rib. If the air escapes readily into the cellular texture, there may be little danger, for it is remarkable how rapidly it is absorbed. But if the air be confined, that side of the chest is not only distended, but the pressure on the mediastinum encroaches on the other cavity, whilst the diaphragm is kept down and impeded in its action. That this was the case in the present instance, the rush of air from the cavity when punctured, and the sudden relief to the respiration are sufficient to prove. Had the cellular membrane only been scarified, as is usually done, the patient must have died suffocated. Here the death must be attributed to the inflammation, the necessary consequence of so extensive an injury.

*Case* 2. M. Harrington, æt. 45, fell on the spikes of a railing, one of which fractured a rib on the right side, and entered the cavity of the chest. On admission into hospital, there was great excitement and anxiety, but no emphysema. V. S. ad ℥xx. the integument closed by adhesive straps, covered by a compress, and the chest bandaged. Calomel and jalap. Active depletion as often as the pulse rose, conducted this case to a speedy and favourable termination. On the sixth day, the opening into the chest was closed, and on the twentieth he left the hospital.

The successful issue of this case must be ascribed to the wound having taken on the adhesive inflammation.

* Mr. Charles Bell and Mr. Shaw. Med. Journ. Sept. 1826.

*Case* 3. (Mr. Shaw.) W. Tateman, æt. 25, fell, June 10th, on a spike, which penetrated near the inferior margin of the right scapula, passed close to the axillary artery, and fractured the third rib, near its junction with the cartilage. There was no emphysema. The pulse 96, respiration difficult.

V.S. ad ℥xviij.—wound dressed with adhesive straps—chest swathed; saline and ant. tart. ad nauseam, ter die. It is unnecessary to follow up the details of this case, but it may be mentioned, that in a few days he lost above eighty ounces of blood; the wound was long in healing; and on the 18th July, he was discharged cured.

In injuries of the chest it is more necessary to bleed *before re-action*, than in any other species of accident, because it is a great object to reduce the volume of blood which is to circulate through the lungs, the functions of which are liable to suffer in more ways than from the inflammation, effusion, extravasation, &c. which succeed re-action. Such cases, therefore, form an exception to the general rule of not bleeding till some degree of re-action takes place in severe accidents. In Mr. Bell's first case, we do not see that a bolder surgery would have offered much prospect of success, on account of the number of ribs broken, and their projection inwards upon the lungs. Any operation that might have removed the projecting points of ribs from their dangerous position, in whatever way effected, would have been a procedure little less formidable than the celebrated operation of Richerand, who sawed away a couple of ribs, and made a large window into the chest, directly over the pericardium!

---

